# Nuclear Receptors in the Control of the NLRP3 Inflammasome Pathway

**DOI:** 10.3389/fendo.2021.630536

**Published:** 2021-02-25

**Authors:** Hélène Duez, Benoit Pourcet

**Affiliations:** Univ. Lille, Inserm, CHU Lille, Institut Pasteur de Lille, U1011- EGID, Lille, France

**Keywords:** nuclear receptors, inflammasome, inflammatory disease, circadian rhythm, NLRP3, therapeutic strategy, inflammation and innate immunity

## Abstract

The innate immune system is the first line of defense specialized in the clearing of invaders whether foreign elements like microbes or self-elements that accumulate abnormally including cellular debris. Inflammasomes are master regulators of the innate immune system, especially in macrophages, and are key sensors involved in maintaining cellular health in response to cytolytic pathogens or stress signals. Inflammasomes are cytoplasmic complexes typically composed of a sensor molecule such as NOD-Like Receptors (NLRs), an adaptor protein including ASC and an effector protein such as caspase 1. Upon stimulation, inflammasome complex components associate to promote the cleavage of the pro-caspase 1 into active caspase-1 and the subsequent activation of pro-inflammatory cytokines including IL-18 and IL-1β. Deficiency or overactivation of such important sensors leads to critical diseases including Alzheimer diseases, chronic inflammatory diseases, cancers, acute liver diseases, and cardiometabolic diseases. Inflammasomes are tightly controlled by a two-step activation regulatory process consisting in a priming step, which activates the transcription of inflammasome components, and an activation step which leads to the inflammasome complex formation and the subsequent cleavage of pro-IL1 cytokines. Apart from the NF-*κ*B pathway, nuclear receptors have recently been proposed as additional regulators of this pathway. This review will discuss the role of nuclear receptors in the control of the NLRP3 inflammasome and the putative beneficial effect of new modulators of inflammasomes in the treatment of inflammatory diseases including colitis, fulminant hepatitis, cardiac ischemia–reperfusion and brain diseases.

## Introduction: The Innate Immune System

Any living organism has to adapt to a specific environment and share common resources with others. To this purpose, organisms may collaborate in a reciprocal relationship from which each one of them benefits for its own survival. On the other hand, organisms may also be subject to threats from pathogenic offenders or from the environment itself, against which they have to defend themselves. The immune system is fundamental to anticipate and to preserve organisms from these threats. For that purpose, a specific system has been developed to allow the detection of two major classes of molecular signals, the pathogen-associated-molecular patterns (PAMPs) and damage-associated molecular patterns (DAMPs) (1). PAMP and DAMP classification appears to be based on their biological sources rather than their chemical structures (1). PAMPs derive from pathogens including microbes and their products, while DAMPs originate from environmental disturbances such as the abnormal accumulation of endogenous compounds and cellular or subcellular damage. A common sensor system, defined as pattern-recognition receptors (PRRs), is able to detect both PAMPs and DAMPs. PRRs which are encoded by innate immune cells such as resident macrophages thus serve as sentinels of environmental changes including the presence of microbes and sterile tissue injury. In addition to PRRs, DAMPs are also detected by non-PRR receptors including receptor for advanced glycation end products (RAGEs), triggering receptors expressed in myeloid cells (TREMs), G-protein-coupled receptors (GPCRs), and ion channel (2). This allows the innate immune system to integrate various deleterious environmental changes to deliver the appropriate response according to nature of the threat (1).

PRRs can be distinguished based on their cellular location and the chemical nature of their ligand. Five main classes have been described: membrane-bound Toll-Like Receptors (TLRs) and C-Lectin Receptors (CLRs), cytoplasmic NOD-like receptors (NLR) and Retinoid acid-inducible gene I (RIG-1)-like receptors (RLRs), and multiple intracellular DNA sensors (CDSs) including cyclic GMP-AMP synthase (cGAS) and absent in melanoma 2 (AIM2) (2). Although TLRs were known to be activated by bacterial wall components such as LPS or proteoglycans, DAMPs including nucleic acids released from damaged cells are able to activate TLR3, TLR7, and TLR9 for instance, while intracellular proteins and extracellular matrix components released after tissue damage are able to induce a TLR2 or a TLR4-dependent signaling cascade (2). In addition, CLRs, usually known to be activated by fungi, are also able to detect lectin-derived compounds such as dendritic cell natural killer lectin group receptor 1 (DNGR1), macrophage-inducible C-type lectin (MINCLE), and Dectin-1 (2). RLRs are able to detect non-self RNA from microbial origin but also inappropriately masked self 5′ppp-RNA such as RNA generated during the unfolded protein response (2). CDSs are able to detect cytoplasmic (cGAS and AIM2) and damaged DNA in the nucleus (AIM2 only) (2). Finally, NLRs, which recognize bacterial compounds such as flagellin, are also able to detect crystals, ATP, amyloid fibers, glucose, or mitochondrial DNA. Therefore, PRRs and non-PPRs are able to sense extracellular and intracellular DAMPs, thus allowing a thorough surveillance of potential threats. Importantly, extracellular signals are considered as low-threat and resolvable problems, while cytosolic signals represent high-threat encounters that may induce pyroptosis, known as an interleukin (IL)-1β and IL-18-triggered cell death program induced by cytosolic PRRs only, mainly inflammasomes (1). When activated by DAMPs, PRRs and non-PRRs then trigger a so-called sterile inflammation, *i.e.* not induced by microbes. Therefore, a sustained activation of these receptors leads to inflammatory diseases including ischemia–reperfusion injury, colitis, systemic lupus erythematous, gout, neurodegenerative diseases, diabetes, atherosclerosis, hepatitis, rheumatoid arthritis, cancer, lung diseases, and gut diseases (2).

Inflammation is characterized by the production of a plethora of secreted immunomodulatory signaling molecules such as histamine, cytokines, chemokines, and lipid derivatives (1). The IL-1 cytokine family is a major cytokine family that includes IL-1α, IL-1β, IL-18, IL-33, IL-36*α*, IL-36*β* and IL-36*γ*. Except for IL-1α, IL-1 cytokines are produced as inactive pro-cytokines and require maturation to biologically active forms by enzymatic cleavage. For instance, pro-IL-1β and pro-IL-18, the most studied IL-1 family members, are processed by the proteolytic activity of Caspase 1, the predominant IL-1 processing protease. Caspase 1 activity is tightly controlled by cytosolic PRR-constituted inflammasome complex. Inflammasomes form the main class of cytosolic PPRs that are activated by diverse exogenous signals including anthrax lethal toxin (NLRP1), bacterial flagellin (NLRC4), double stranded DNA (AIM2), toxin-induced modifications of Rho-GTPase (Pyrin). Unlike other inflammasomes, the nucleotide-binding domain (NOD)-, Leucine-rich repeat (LRR)- and pyrin domain containing protein 3 (NLRP3) inflammasome is not only activated by microbial and environmental molecules but also by several metabolic products including ATP, cholesterol crystals and *β* amyloid fibers. In this regard, NLRP3 is unique because it is able to sense a wide range of threats. NLRP3 is therefore a central PAMPs and DAMPs sensor whose erratic activation leads to numerous NLRP3-driven diseases.

## The NLRP3 Inflammasome

The NLRP3 inflammasome was first identified in the cryopyrin-associated periodic syndrome (CAPS) and was later recognized to be involved in many other inflammatory/metabolic diseases including gout, atherosclerosis, type 2 diabetes, non-alcoholic fatty liver diseases (NAFLD), colitis, and neurodegenerative diseases such as Alzheimer and Parkinson diseases. The NLRP3 inflammasome is not only expressed by leucocytes (macrophages, dendritic cells, neutrophils) but also by hepatocytes, neurons, endothelial cells, cardiomyocytes, and pancreatic beta cells (3).

### Structure

The NLRP3 inflammasome is a supramolecular organizing center (SMOC) which consists of a sensor (NLRP3), an adaptor (Apoptosis-associated speck-like protein containing a Caspase recruitment domain (ASC) encoded by *PYCARD*), and an effector (Caspase 1) (4). NLPR3 contains an amino terminal pyrin domain (PYD) involved in protein–protein interaction, a central oligomerization domain (NOD, nucleotide-binding and oligomerization domain, NACHT) with an ATPase activity involved in the self-association and function of NLRP3 and a carboxy terminal leucin-rich repeat (LRR) domain inducing the autoinhibition of NLRP3 by folding back onto the NACHT domain (4). Apart from an N_ter_ PYD domain, ASC also includes a C_ter_ caspase recruitment domain (CARD) that plays a role of adaptor platform for the pro-Caspase 1 protein through a CARD–CARD domain interaction. Caspase 1 structure also includes a central catalytic domain (p20) and a C_ter_ small catalytic subunit (p10) (4).

### Function

**Figure 1:**
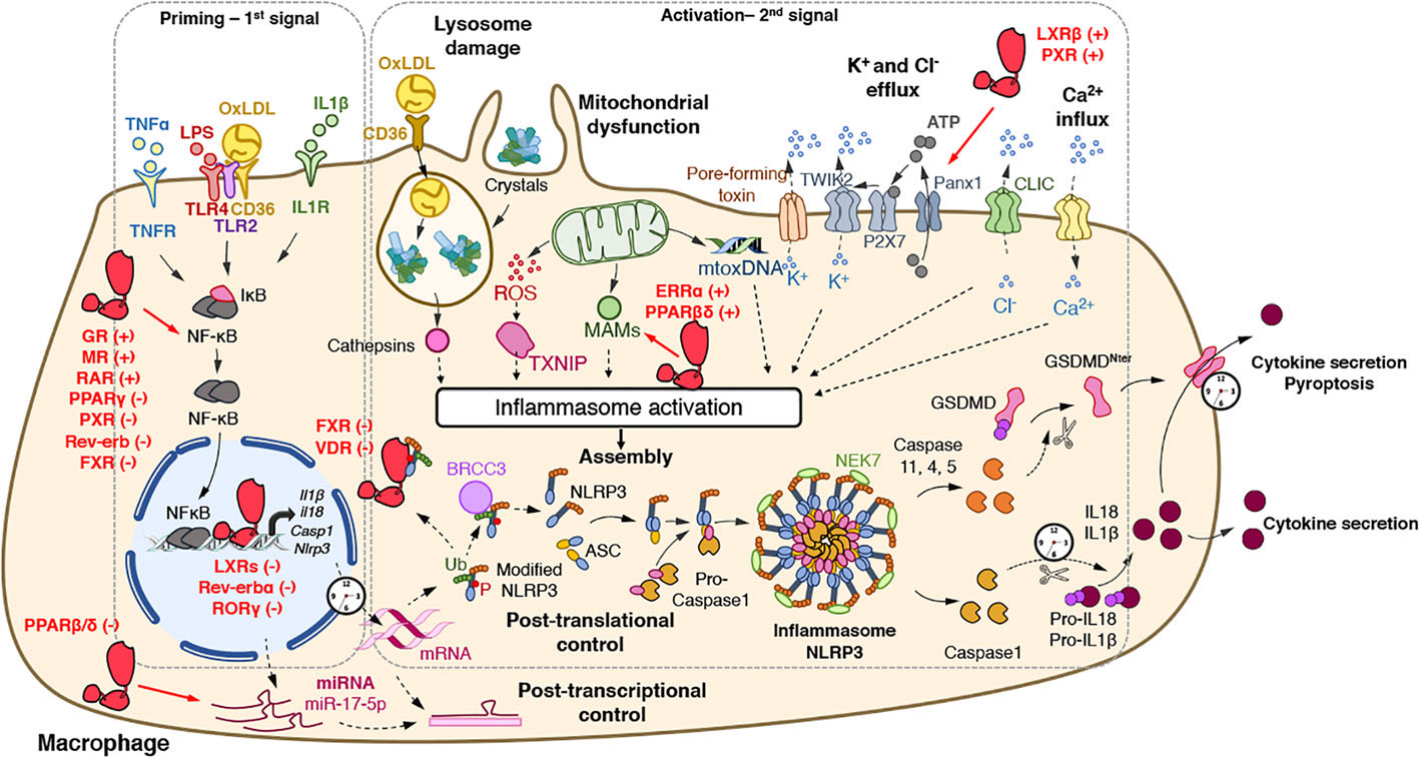
Regulatory activities of nuclear receptors on the NLRP3 inflammasome priming and activation steps. The priming (first step) of the NLRP3 inflammasome requires the binding and activation of PRRs (TLRs,…) by PAMPs such as LPS, cytokines or ox-LDL, resulting in the transcription of the NLRP3 inflammasome components. Its activation (second step) is the result of recognition of PAMPs (such as the bacterial pore-forming toxin nigericin) or DAMPs which are released by damaged or dying cells (such as ATP) following injury or metabolic imbalance (such as mtROS), or accumulate in tissues (such as crystals). These lead to lysosomal damage, mitochondrial damages (exposition of cardiolipin, mtDNA) which ultimately modify ion (K^+^, Ca^2+^) fluxes. Upon this two-step process, the NLRP3 inflammasome assembles, caspase 1 is activated, Gasdermin-D and pro-IL-1β and pro-IL-18 are cleaved, leading to mature cytokines secretion and cell death by pyroptosis. The activity of nuclear receptors on each step is indicated when appropriate. ASC, apoptosis-associated speck-like protein containing a CARD domain; ATP, adenosine triphosphate; BRCC3, Lys-63-specific deubiquitinase BRCC36; casp, caspase; CLIC, chloride intracellular channels; DAMPs, damage-associated molecular patterns; GSDMD, gasdermin-D; IL, interleukin; IL1R, interleukin-1 receptor; LPS, lipopolysaccharide; MAM, Mitochondria-associated ER membranes; mtoxDNA, mitochondrial oxidized DNA; NFκB, nuclear factor-kappa B; NLRP3, nucleotide-binding, LRR and PYD domains-containing protein 3; Ox-LDL, oxidized low-density lipoproteins; P, Phosphate; PAMPs, pathogen-associated molecular patterns; Panx1, Pannexin-1; PRRs, Pattern Recognition Receptors; ROS, reactive oxygen species; P2X7, purinergic receptor P2X7; TLR, Toll-like receptor; TNF, tumor necrosis factor; TNFR, tumor necrosis factor receptor; TWIK2, two-pore domain weak inwardly rectifying K^+^ channel; TXNIP, Thioredoxin-interacting protein Ub, ubiquitin. (+): activates; (−): inhibits.

Upon stimulation, NLRP3 oligomerizes through homotypic interactions between NACHT domains of two NLRP3 proteins and the subsequent recruitment of ASC through PYD-PYD interactions (**Figure 1**). Then, helical ASC filaments nucleate and associate to form macromolecular ASC specks (5–7) (**Figure 1**). Finally, assembled ASC recruits pro-caspase 1 in a CARD-dependent manner that enables the proximity-driven self-cleavage of pro-caspase 1 to generate p33 (comprising the CARD and the p20 domains) and p10, which remains bound to ASC and becomes proteolytically active (**Figure 1**). Further processing then triggers the release of the p20 and p20–p10 complex from ASC. The p20–p10 complex is unstable in the cells, thus terminating its protease activity (8). Beyond the classical representation of NLRP3 inflammasome assembly, it has recently been demonstrated that the NIMA-related kinase 7 (NEK7) oligomerizes with the LRR domain of NLRP3 into a complex by bridging the gaps between adjacent NLRP3 subunits to mediate NLRP3 oligomerization that is essential for ASC speck formation and caspase 1 activation (9, 10) (**Figure 1**). Strikingly, NEK7 is specific to NLRP3 and does not interact with other inflammasomes such as NLRC4 (11). Regulation of NEK7-NLRP3 assembly is induced by ATP-driven potassium efflux (12) but also in a K^+^-efflux independent manner (13) and by reactive oxygen species (ROS) production (9). Activated-Caspase 1 is then able to process pro-IL-1β and pro-IL-18 into mature and functional IL-1β and IL-18 (**Figure 1**).

In addition to the regulation of pro-inflammatory cytokine maturation, the NLRP3 inflammasome is also involved in the control of pyroptosis, defined as a rapid and inflammatory form of programmed cell death. Pyroptosis actually results from the cleavage of Gasdermin D (GSDMD) by inflammatory caspases including caspases 1, 4, 5, or 11 (14–16) (**Figure 1**). GSDMD possesses an N_ter_ cell death domain (GSDMD^NTerm^), a central short region, which links to a C_ter_ auto-inhibition domain. Caspase 1 cleaves pro-GSDMD, thereby removing the auto-inhibition domain, thus alleviating the inhibition on the cell death domain (**Figure 1**). GSDMD^NTerm^ then binds to phosphatidylinositol phosphate and phosphatidylserine in the inner leaflet of the cell membrane, oligomerizes, and inserts into the plasma membrane, thus forming a pore of 16 symmetrical protomers that kill the cell (17).

### Activation of the NLRP3 Inflammasome

The activation of the NLRP3 inflammasome, as most inflammasomes, is tightly controlled by a two-step process (4) (**Figure 1**). A priming step is required to increase gene and protein expression of its components in order to sense stimuli and become activated. Once the cytoplasmic levels of NLRP3 mRNA reach an activating threshold, inflammasome assembly can be triggered by a secondary signal. This activation step initiates the NLRP3 SMOC assembly that promotes Caspase 1 autocatalytic activity and its subsequent maturation.

#### Priming the NLRP3 Inflammasome

The priming step has two main purposes: the transcriptional induction of the inflammasome complex components NLRP3, Caspase 1, IL-1β, and IL-18 and the induction of post-translational modifications of NLRP3 (**Figure 1**). The first one can be induced through the recognition of various PAMPs and DAMPs by PRRs such as TLRs and NLRs including NOD1 and NOD2 or cytokine receptors (*e.g.* TNFR) whose activation promotes nuclear factor *κ*B (NF-*κ*B) transcriptional factor activation and the subsequent induction of *Nlrp3* and *Il1β* gene transcription (**Figure 1**).

In addition to classical TLR ligands, disruption of metabolic homeostasis has also been involved in the NLRP3 inflammasome priming. For instance, NLRP3 mediates trained immunity following western diet feeding (18), suggesting that a lipid-rich diet potentializes the NLRP3-mediated response to pro-inflammatory stimuli. Accordingly, oxidized LDL (oxLDL), but also islet amyloid polypeptide (IAPP) and Alzheimer Disease beta amyloid peptides (Aβ_1–42_), induce *Nlrp3* and *Il1β* gene expression, and thus the priming of this pathway in a CD36-TLR2-TLR4 heterotrimer-dependent manner in bone marrow-derived macrophages (BMDM) (19, 20) (**Figure 1**). Finally, cholesterol crystal-induced release of Neutrophils Extracellular Traps (NETs) from neutrophils is also able to prime NLRP3 in macrophages through the activation of several TLRs (21).

In addition to transcriptional regulation, the stability of mRNA of inflammasome components such as NLRP3, Casp1, and Casp8 can be controlled at the post-transcriptional level by miRNA [see (22) for review]. For instance, miR-223-3p negatively regulates the NLRP3 inflammasome by targeting the 3′-untranslated region (UTR)-binding sites of NLRP3 mRNA in myeloid cells (23). In addition, miRNAs can also target the mRNA of upstream regulators of the NLRP3 inflammasome including TXNIP, TRAF6, and SOD2 (11). As an example, miR-17-5p decreases TXNIP mRNA stability and NLRP3 activation in insulin producing cells and in the brain, thus inhibiting NLRP3 pathway activation (24, 25) (**Figure 1**). Accordingly, altered expression of several miRNAs is associated with the development of numerous NLRP3-driven diseases such as rheumatoid arthritis (26), multiple sclerosis (27), and systemic lupus erythematosus (28, 29).

While this transcriptional priming allows the production of NLRP3 pathway components, additional mechanisms are necessary to maintain NLRP3 in an inactive but poised configuration to rapidly respond to an activation signal. The second function of priming is then the induction of rapid transcription-independent mechanisms that regulate NLRP3 stability in order to rapidly progress from this poised state to an active one. Such non-transcriptional mechanisms are mainly classical post-translational modifications including ubiquitination, phosphorylation and SUMOylation (**Figure 1**) [see for review (30)]. For instance, ubiquitination of NLRP3 by FBXL12, TRIM1, ARIH2 or the dopamine-induced E3 ligase MARCH7 promotes the proteasomal degradation of NLRP3 in resting macrophages (30), whereas deubiquitylation of NLRP3 LRR domain on K63 by BRCC3 triggers ASC oligomerization and inflammasome activation (31, 32) (**Figure 1**).

#### Activation

The NLRP3 inflammasome is unique as it can assemble in response to a wide range of stimuli with various chemical properties. These include exogenous molecules of various origins such as environmental particulates (silica crystals) or pathogens. In addition, many endogenous molecules that abnormally accumulate are able to activate the NLRP3 inflammasome. This abnormal accumulation usually reflects tissue damage or metabolic dysfunction, which are thus sensed by NLRP3. For instance, under physiological conditions, LDLs normally circulate in blood. When LDL level abnormally increases in the context of dyslipidemia and when the vascular endothelium is damaged, LDLs infiltrate into the vascular wall, are eventually oxidized and trigger macrophage recruitment as seen in atherogenesis. CD36-mediated uptake of oxLDLs by macrophages contributes to the formation of intracellular cholesterol crystals and leads to the subsequent activation of NLRP3 (19) (**Figure 1**). Likewise, while normal extracellular ATP levels are harmless, tissue damage or cell death increases extracellular ATP levels acting as NLRP3-activating DAMPs. NLRP3 activation is often due to cellular stress resulting in lysosomal destabilization, ion flux imbalance, and redox potential alteration.

##### Lysosomal Damage

Crystals (cholesterol, urea, hydroxyapatite crystals) or fibrillar protein aggregates (*β*-amyloid, IAPP) can be phagocytosed by immune cells and then traffic toward lysosomes. These crystals often lead to lysosomal damage resulting in the release of proteases such as cathepsins (**Figure 1**). Although lysosomal disruption appears as a critical step for NLRP3 activation (33), downstream mechanisms between lysosome alteration and activation of the NLRP3 inflammasome still need to be unequivocally identified. Lysosome-released cathepsin B was considered for long as an essential trigger of NLRP3 activation (33). Nevertheless, the use of a broad spectrum of cathepsin inhibitors and individual knock-out experiments of several cathepsins confirmed that NLRP3 activation however, relies on several cathepsins that may exert redundant activities (34, 35). Importantly, Leu-Leu-OMe-induced lysosomal damage enhances K^+^ and Ca^2+^ efflux that may account for lysosomal damage-controlled NLRP3 activation (36).

##### Ion Fluxes

Ion fluxes are important regulators of NLRP3 inflammasome activation. Changes in ion homeostasis such as increased intracellular Ca^2+^ levels as well as decreased intracellular K^+^ and Cl^−^ levels also appear to play a pivotal role in NLRP3 activation (**Figure 1**). Lower extracellular concentrations of K^+^ compared to intracellular K^+^ concentrations are sufficient to induce K^+^ efflux and to promote NLRP3 activation while high levels of extracellular K^+^ prevent its activation in THP1 cells and BMDM (37, 38). In addition, nigericin, a K^+^ ionophore, as well as the ATP-mediated activation of P2X purinoceptor 7 (P2rx7), a ligand-gated ion channel, promotes K^+^ efflux-dependent IL-1β maturation (39–41) (**Figure 1**). Interestingly, P2rx7 does not directly control K^+^ efflux, but instead, promotes Ca^2+^ and Na^2+^ influx after ATP stimulation and coordinates with the K^+^ channel two-pore domain weak inwardly rectifying K^+^ channel (TWIK2), which mediates K^+^ efflux (42) (**Figure 1**).

Interestingly, K^+^ efflux must be associated with Ca^2+^ influx to promote mitochondrial-mediated ROS production (43), where Ca^2+^ influx appears critical for NLRP3 activation (44, 45) (**Figure 1**). At the molecular level, CHOP, a transcription factor activated during ER stress, promotes Ca^2+^ release from the ER, thus stimulating the calcium-sensing receptor (CASR) and promoting NLRP3 assembly (44). K^+^ efflux also controls Ca^2+^ release from the ER demonstrating the interconnection between the different activating signals (43, 44).

In addition to K^+^ efflux and Ca^2+^ influx, Cl^-^ flux has also been demonstrated to activate NLRP3 (**Figure 1**). Indeed, while low extracellular Cl^−^ enhances ATP-induced IL-1β secretion, high extracellular Cl^−^ concentration or Cl^−^ channel blockers inhibit NLRP3 activation (46, 47). Two recent reports demonstrated that chloride intracellular channels (CLICs), especially CLIC1 and CLIC4 mediate NLRP3 activation by promoting Cl^−^ efflux downstream nigericin-induced K^+^ efflux and mitochondrial ROS production, which promotes CLIC translocation to the plasma membrane (46, 47) (**Figure 1**). Interestingly, K^+^ seems to drive NLRP3 oligomerization, probably in a NEK7-dependent manner (12, 48), while Cl^−^ efflux is prone to induce ASC polymerization (48). Finally, although ion fluxes were shown to control NLRP3 assembly and activation, the link between ion fluxes and the inflammasome activation remains to be identified.

##### ROS Production and Mitochondrial Dysfunction

Since ROS scavengers attenuate NLRP3 activation, the generation of ROS was considered a common cellular response critical for NLRP3 activation (49). Although the source of NLRP3-activating ROS was controversial, the inhibition of the lysosomal NADPH oxidase did not alter NLRP3 activation in mouse and human cells, thus suggesting an alternative source of NLRP3-activating ROS, likely the mitochondria (33, 50, 51). After stimulation with various NLRP3 activators, mitochondrial ROS (mtROS) altogether with Ca^2+^, contribute to the rapid release of mtDNA into the cytosol (52) where it is eventually oxidized (53). Oxidized mtDNA then specifically interacts with NLRP3 and activates the inflammasome (53) (**Figure 1**). In addition, mtROS promotes Thioredoxin-interacting protein (TXNIP)-NLRP3 interaction involved in NLRP3 expression (54) (**Figure 1**).

Notably, NLRP3 is mainly localized at the membrane surface of ER in unstimulated cells (49). However, in the presence of MSU, nigericin or alum, mtROS production leads to the rapid relocation of NLRP3 and cardiolipin at the mitochondria outer membrane and promotes K^+^ efflux (49). Then, the ASC adaptor accumulates at Mitochondria-associated ER membranes (MAMs) where the NLRP3-ACS complex is formed (49). In addition, NLRP3 may also interact with mitochondrial antiviral-signaling protein (MAVS), which is another mitochondrial outer MAM (55–57). In this context, mitofusin 2 can also be found in the outer mitochondrial membrane, the ER and MAM. Mitofusin 2 plays an important role in NLRP3 activation during RNA viral infections since it interacts with MAVS to support the relocation of NLRP3 to the mitochondria (58) (**Figure 1**).

#### Alternative Inflammasome Activation and Non-Canonical NLRP3 Activation

In addition to the classical/canonical NLRP3 inflammasome activation, an alternative NLRP3 activation process has been identified in which LPS alone is sufficient to induce inflammasome activation without the involvement of another second activator (59). This signaling pathway relies on a cascade involving TLR4, TIR domain-containing adapter molecule 1 (TRIF), RIPK1, FADD and caspase 8 that finally promotes NLRP3 activation. Interestingly, in addition to LPS, the pro-atherogenic apolipoprotein ApoC3 is able to trigger TLR2 and TLR4 heterodimerization and promotes the alternative activation of NLRP3 (60), thus mirroring the effect of oxLDL in the canonical activation of NLRP3. Strikingly, the alternative inflammasome activation is characterized by its independency on K^+^ efflux and the absence of pyroptosome formation and pyroptosis. Then, this pathway is likely involved in the control of cytokine secretion without affecting cell viability.

In addition to caspase 1, cytosolic gram negative bacteria-derived LPS may also be sensed independently of TLR4 signaling by human caspases 4 and 5, and mouse caspase 11, to induce the non-canonical NLRP3 inflammasome (61, 62). In this pathway, Caspase-4/5/11 promote pyroptosis by processing pro-GSDMD and pannexin-1, a protein channel that releases ATP from the cell. This extracellular ATP then activates P2xr7 to promote K^+^ efflux and NLRP3 activation (63, 64).

## Nuclear Receptors

In addition to the above-described regulators, priming and activation processes are also controlled by nuclear receptors (NRs), a subclass of transcription factors. Although numerous studies have reported this alternative activation pathway, such regulatory processes are rarely mentioned. We provide here the first review of the literature describing how these lipid-regulated receptors control both priming and activation processes in the context of different NLRP3-driven diseases. We will also describe in which pathophysiological contexts this regulation has been reported and how the pharmacological modulation of these NRs prevents the progression of NLRP3-driven diseases. Finally, we will discuss also the role of NLRP3 in NR regulation.

### Nuclear Receptors: Generalities

**Figure 2:**
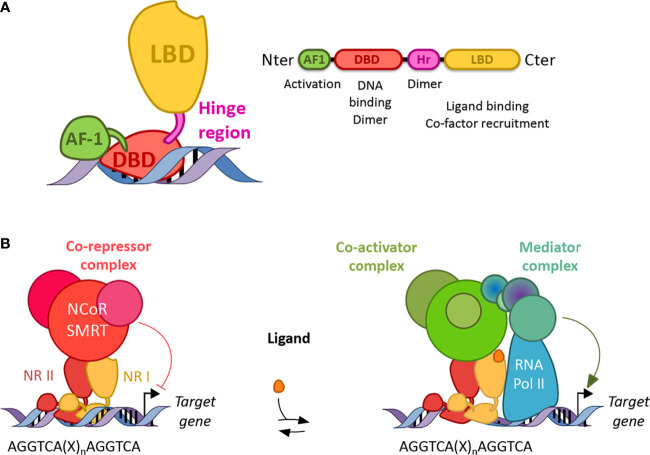
Structure and function of nuclear receptors. **(A)** Canonical structures of nuclear receptors. Nuclear receptors are composed of a N terminal activation function domain whose activity is independent of ligand binding, a DNA binding domain (DBD), a hinge region (Hr) and a ligand binding domain (LBD). Their respective activity is mentioned accordingly. Dimer: dimerization **(B)** Nuclear receptors work as homo or heterodimers which bind a response element present in the promoter of their target genes. Response elements are composed of two AGGTCA half-sites separated by one to four nucleotides (X). In the absence of ligand, NRs (except class I) preferentially bind co-repressor and inhibit gene transcription. In the presence of a ligand, co-repressors are degraded by the proteasome and co-activators are recruited, which then allows the binding of a mediator complex and the ARN polymerase II.

Discovered in the mid-80s, NRs represent a superfamily of structurally conserved ligand-dependent transcription factors that regulate gene expression (65–67). The nuclear receptor superfamily can be sub-divided into four classes based on their ligand- and DNA-binding properties and on the nature of their partner (68). NRs usually work as homo- or heterodimers, which bind to a specific response element composed of two AGGTCA half-sites separated by one to four nucleotides in the promoter of target genes (**Figure 2**). These half-sites are organized either as a palindromic sequence or a direct repeat. The first class, mostly classical steroid hormone receptors, is probably the best characterized and consists of nuclear hormone receptors such as Androgen Receptor (AR), Glucocorticoid receptor (GR), Estrogen receptors (ERs: ER*α*, ER*β*), Mineralocorticoid Receptor (MR), Progesterone Receptor (PR). These NRs work as homodimers and are recruited to a palindromic arrangement of core recognition motifs. The second class consists of so-called adopted receptors that were initially identified as orphan receptors meaning without known ligands, but subsequent studies characterized naturally occurring ligands and determined their physiological roles. Its members encompass eicosanoid and fatty acid receptors Peroxisome Proliferator-Activated Receptors (PPARs: PPAR*α*, PPAR*β*/*δ*, PPAR*γ*), the oxysterol receptors Liver X Receptors (LXRs: LXR*α* and LXR*β*), Thyroid hormone Receptors (TRs: TR*α*, TR*β*), the Retinoic Acid Receptors (RARs: RAR*α*, RAR*β*, RAR*γ*), the Vitamin D3 Receptors (VDR), the xenobiotic receptor Pregnane X Peceptor (PXR). NRs from class II heterodimerize with one of the Retinoid X Receptors (RXRs: RXR*α*, RXR*β* or RXR*γ*) and are recruited to a response element organized in two-half sites in tandem repeat (69–71). The third NR class is composed of adopted receptors such as RXRs, the heme receptors Rev-erb (Rev-erb*α* and Rev-erb*β*), fatty acid receptor Human Nuclear Factor 4 (HNF4*α*, HNF4*γ*) and orphan receptors such as Chicken Ovalbumin Upstream Promoter Transcription Factor (COUP-TFI, COUP-TFII). The NRs from this third class act as monomers or homodimers bound on direct repeat response elements. Finally, the fourth class is made of orphan nuclear receptors such as Estrogen Related Receptors (ERR*α*, ERR*β*, ERR*γ*), Retinoid-related Orphan Receptors (ROR*α*, ROR*β*, ROR*γ*), Nurr1, NOR1, Nurr77 and the steroidogenic factor 1 SF-1. Therefore, NRs represent a crucial superfamily of transcriptional factors whose transcriptional activity may be modulated by specific natural or synthetic ligands, identifying NRs as promising therapeutical targets in numerous diseases, and especially in NLRP3-driven diseases as described below.

#### Structure and Molecular Functions of Nuclear Receptors

NRs consist of modular domains, including a variable amino N-terminal activation domain (AF-1), a highly conserved DNA-binding domain (DBD), a conserved hinge region linking the DBD with the conserved ligand-binding domain (LBD) (72) (**Figure 2A**). The DBD mediates the specific recruitment of NR monomers, homodimers, heterodimers to their DNA response element and is involved in the dimerization of NRs with their partner altogether with the hinge region and the LBD. In addition, the LBD mediates ligand-dependent interactions with transcriptional co-activators such as p300/CBP or co-repressors such as NCoR or SMRT (**Figure 2**). These interactions are controlled, at the structural level, by ligand-dependent conformational changes in the last *α*-helix 12 (*α*H12) of the LBD known as AF2 (73). In the absence of a ligand, co-repressors are preferentially bound to NRs, especially those of class II, while ligand binding induces a conformational change of the *α*H12 helix which then triggers the release of co-repressors, allowing co-activator binding (**Figure 2B**). If several NRs, especially those of the class II including PPARs, LXRs, RARs are then able to bind target genes in the absence of a ligand and recruit co-repressors to actively repress gene expression, class I steroid hormone receptors are usually sequestered into the cytoplasm in the absence of ligands and are translocated into the nucleus to bind their target genes in the presence of a ligand. Finally, the Rev-erb subfamily, Rev-erb*α* and Rev-erb*β*, lacks the *α*H12, which then prevents the recruitment of co-activators (74, 75). Instead, although Rev-erbs are able to recruit co-repressors and actively repress gene expression in the absence of a ligand, ligand binding enhances co-repressor recruitment and the transcriptional activity of Rev-erbs to further inhibit the expression of their target genes (74, 75). Their transcriptional activity might be regulated by post-translational modifications including phosphorylation, ubiquitination, and SUMOylation (76–85).

#### Nuclear Receptors and the Innate Immune System

NRs are involved in the control of numerous physiological activities including metabolism (86, 87), reproduction (88, 89), cell cycle (90), vasculature (91, 92), brain activity (93, 94), circadian rhythm (95–97) and immunity (98–103). NRs have then been widely implicated in the control of inflammatory processes and the control of immune cell activity (99). In macrophages, many NRs display anti-inflammatory activities by quenching the NF-*κ*B dimer into the cytoplasm (99). For instance, GR inhibits the expression of TNFα and COX2. In addition, iNOS expression is inhibited by both PPAR*γ* and GR, while TLR4 expression is dampened by Rev-erb*α* and PPAR*γ* (78, 104). In the same manner, IL-6 expression is reduced by both GR and Rev-erb*α* (99, 105). Interestingly, LXR*α* is able to induce TLR4 in human macrophages only, emphasizing the species-specificity of such regulatory pathway and also induces its own negative regulatory loop by enhancing Rev-erb*α* expression to avoid TLR4 lasting expression (106). In addition, nuclear receptors such as PPAR*γ* (107), LXR*α* (101, 108, 109), Nurr77 (110) and Rev-erb*α* (104, 111) also control the skewing of pro-inflammatory macrophages toward anti-inflammatory macrophages. Finally, nuclear receptors including LXR (112), GR (113), and Rev-erb (105, 114) regulate macrophage recruitment by controlling the production of adhesion molecules or the secretion of chemokines such as Monocyte Chemoattractant Protein 1 (MCP1).

## Nuclear Receptors in the Priming of NLRP3

### Nuclear Receptors Control NF-κB-Dependent Regulation of NLRP3 Inflammasome

Many NRs have been shown to interact with the NF-*κ*B complex and to inhibit this pathway either by directly interacting with the NF-*κ*B complex in the cytoplasm, a mechanism known as quenching (98, 99), or by preventing the polyubiquitination of the IKK complex, which then promotes NF-*κ*B inhibition (98). However, although these regulatory processes are known, only few studies demonstrate the direct link between NR-controlled NF-*κ*B pathway and NLRP3 priming. For instance, dexamethasone, a GR synthetic ligand, and cortisol treatments in human THP1 macrophages and in BMDMs induce the expression of NLRP3 mRNA and proteins in a GR-dependent manner but not those of *Casp1* and *Il1β* (115) (**Figure 1**). Nevertheless, glucocorticoids enhance the secretion of mature IL-1β by these cells (115), thus demonstrating the ability of glucocorticoids to set up an active NLRP3 inflammasome pathway. Although the molecular mechanisms involved therein were not investigated in this report, this regulatory effect may be due, at least partially, to the activation of the NF-*κ*B pathway. Indeed, dexamethasone as well as chronic stress, which triggers the production of cortisol, induce the NF-*κ*B pathway in hippocampal neuroinflammation and depression-like behavior (116). In addition to GR, PXR agonists altogether with PXR overexpression induce NLRP3 and NLRP2 mRNA levels in endothelial HUVEC cells (117) (**Figure 1**). Interestingly, oxLDL has been shown to induce NLRP3 expression in a LOX1- and NF-*κ*B-dependent manner (118). LOX1 is the main endothelial oxLDL receptor, whose stimulation by oxLDL induces NF-*κ*B pathway (119), a mechanism reminiscent to the CD36-dependent one in macrophages (19). Interestingly, statins inhibit the activated NF-*κ*B pathway and NLRP3 inflammasome by oxLDL in vascular endothelial cells through a PXR-dependent mechanism as well (118). Intriguingly, PXR blocks NF-*κ*B binding in oxLDL-primed HUVEC, thus suggesting that PXR activation inhibits NLRP3 activation (118). Furthermore, epleronone-mediated inhibition of MR suppresses the expression of NLRP3 and Caspase 1 both in the liver and epididymal white adipose tissue (eWAT) (120) (**Figure 1**). However, whether these epleronone-mediated effects on NLRP3 pathway are dependent on MR remain to be confirmed. For instance, it is unknown whether MR-response elements are present in the promoter of inflammasome component coding genes. Accordingly, MR knock-down impairs aldosterone regulatory effect on IL-1β expression in LPS-stimulated BMDM, but this effect was likely due to an inhibition of NF-*κ*B phosphorylation instead of a direct effect on NLRP3 gene expression (120). It is noteworthy that increased MR expression is associated with an increase in NLRP3 expression and altered microglia phenotype in hippocampus from spontaneously hypertensive rats (121). However, the actual functional impact of MR in this process needs further investigation to prove the implication of MR in this context. Furthermore, NRs such as RXRs and RAR are activated by retinoic acids including 9-*cis*-retinoic acid (9-*cis*-RA) and all-*trans*-retinoic acid (ATRA). Interestingly, human LPS-primed macrophages treated with ATRA exhibit elevated NLRP3 RNA and protein levels associated with an increase in caspase 1 and pro-IL-1β maturation. At the molecular level, ATRA alone induces NLRP3 expression and enhances LPS-induced NRLP3 and IL-1β mRNA levels by upregulating the phosphorylation of I*κ*B, ERK, and p38 (122). Therefore, stimulation of GR, MR, PXR, and RAR induces NLRP3 priming. Besides, PPAR*γ* also controls the NF-*κ*B-dependent NLRP3 priming in different contexts including astrocytes and retinal ischemia/reperfusion (123, 124). Here, IL4-activated PPAR*γ* inhibits NLRP3 protein levels in an NF-*κ*B-dependent manner in High Mobility Group Box-1 (HMGB-1)-stimulated astrocytes (124), while treatment with pioglitazone, a PPAR*γ* agonist, ameliorates retinal ischemia/reperfusion-mediated inflammatory response by suppressing NLRP3 activation in an NF-*κ*B-dependent manner (123) (**Figure 1**). Furthermore, GW4004-mediated activation of FXR also inhibits the expression of TLR4 and Myd88 in ileum (125). The gene expression of the NLRP3 inflammasome pathway components was altered accordingly, although the direct impact of GW4004 on NF-*κ*B activation was not reported in this context (125). Finally, in addition to PPAR*γ*, Rev-erb*α* may also inhibit NLRP3 priming, at least partially, *via* the inhibition of p65 expression in mouse RAW264.7 macrophage cell line (126). Accordingly, modulation of Rev-erb activity revealed that Rev-erbs may inhibit p65 and I*κ*B phosphorylation in RAW264.7 cells thus inhibiting NF-*κ*B activity (127). Together these data indicate that PPAR*γ* and Rev-erb*α* may inhibit NK-*κ*B-dependent NLRP3 priming (**Figure 1**). Overall, as many NRs including the Constitutive Androstane Receptor (CAR) (128, 129) and PPAR*α* (130, 131), have been demonstrated to control the NF-*κ*B pathway (103), it could be anticipated that they may also be involved in NF-*κ*B-dependent NLRP3 priming processes, although this still needs to be proven.

### Nuclear Receptors Directly Regulate NLRP3 Priming

NRs are also able to directly control NLRP3 transcription. For instance, Rev-erb*α*, a transcriptional repressor, is directly recruited to four distinct Rev-erb Response Elements (RevRE) into the *Nlrp3* gene promoter and actively inhibits *Nlrp3* expression in both human and mouse primary macrophages (132) (**Figure 1**). Furthermore, the deletion of ROR*γ* or the use of a ROR*γ* inverse agonist decreases NLRP3 mRNA and protein levels, which is associated with a reduction of IL-1β secretion in LPS-primed BMDM (133). ROR and Rev-erb share the same consensus sequence allowing them to bind the same RORE/RevRE response elements (**Figure 1**). Accordingly, ROR*γ* was found to be recruited to the same Rev-erb*α* sites in the *Nlrp3* promoter (132, 133). Finally, although poorly investigated, NRs also control *Nlrp3* mRNA post-transcriptional stability through the regulation of miRNA. Indeed, the PPAR*β*/*δ* agonist, GW0742, significantly reduces the number of activated pro-inflammatory microglial cells after hypoxia–ischemia in neonatal rat brain (134). This effect is mainly due to a decrease in TXNIP, NLRP3, IL-6 and TNFα (134). At the molecular level, the PPAR*β*/*δ* antagonist GSK3787 and the miR-17-5p inhibitor abolish GW0742 effect, thus demonstrating the dependency of GW0742 on the PPAR*β*/*δ*-miR-17-5p axis (134) (**Figure 1**). However, the identification of the precise mechanisms by which PPAR*β*/*δ* controls the regulation of miR-17-5-p still needs further investigations. It is not excluded either that other NRs may regulate miRNA expression implicated in the post-transcriptional regulation of *Nlrp3* mRNA stability.

## Nuclear Receptors Regulate the NLRP3 Activation Step

In addition to NLRP3 priming, nuclear receptors are also able to control NLRP3 activation, *ie* the second step of NLRP3 regulation. For instance, deletion of *Rev-erbα* increases nigericin- and ATP-induced ASC speck formation in mouse primary macrophages, thus suggesting that Rev-erb*α* prevents NLRP3 inflammasome assembly and its activation (132). However, the underlying mechanisms still need to be uncovered, and it cannot be excluded that this effect on NLRP3 activation reflects only the increase of *Nlrp3* gene expression triggered after Rev-erb*α* deficiency. However, because Rev-erb*α* regulates mitochondrial function and autophagy processes in skeletal muscle (135), we may speculate that the inhibition of NLRP3 assembly by Rev-erb*α* could be mediated by a decrease in ROS production and an enhancement of mitochondrial function.

Interestingly, the bile acid receptor FXR is also able to physically interact with NLRP3 and Caspase1 thus inhibiting NLRP3 activity (136) (**Figure 1**). In addition, bile acids behave as DAMPs and inhibit the priming and activation of the NRLP3 inflammasome in the context of cholestatic and septic mice (136). At the molecular level, bile acids induce a prolonged Ca^2+^ influx and activate NLRP3 synergistically with ATP administration (136). It is noteworthy that these effects are independent of ROS production and K^+^ efflux (136). In this context, FXR deletion increases endotoxemia sensitivity while FXR overexpression increases mice resistance to endotoxemia, thus suggesting an FXR-independent effect of bile acids action in sepsis (136). Such FXR-independent effect of bile acid on NLRP3 inflammasome may be mediated by the membrane receptor Takeda G coupled Receptor 5 (TGR5), another bile acid receptor. Indeed, treatment of BMDM with bile acids suppresses LPS/Nigericin-mediated NLRP3 activation in a TGR5-cAMP-PKA dependent by inducing NLRP3 ubiquitination and phosphorylation (137–140).

Furthermore, vitamin D enhances VDR-mediated inhibition of NLRP3 activation (141). Indeed, vitamin D3 (VitD3) inhibits NLRP3 activation in LPS-primed mouse peritoneal macrophages in the presence of nigericin, MSU or alum (142). In addition, vitD3 dampens ASC speck formation by preventing the NLRP3/NEK7 interaction (142). Interestingly, vitD3 also promotes NLRP3 ubiquitination. Indeed, the LBD of VDR is able to physically interact with the NACHT-LRR domain of NLRP3 thus inhibiting the association of NLRP3 with BRCC3 and preventing NLRP3 deubiquitination (141) (**Figure 1**). Particularly, VDR has been shown to prevent NLRP3 modification on K63 and its subsequent activation (141). Finally, vitD3 also increases VDR-controlled UCP2 expression thus inhibiting ROS accumulation in LPS-primed peritoneal macrophages (141). Altogether, VDR inhibits NLRP3 inflammasome by favoring NLRP3 ubiquitination, preventing NLRP3 assembly and reducing ROS-mediated NLRP3 activation.

LXRs have also been shown to modulate the NLRP3 pathway. In colon cancer cells for instance, LXR*β* activates NLRP3 inflammasome by inducing Pannexin1-dependent ATP release and autocrine P2x7R activation, which in turn leads to anti-tumoral effect of LXR agonists (143) (**Figure 1**). By contrast, LXRs have also been shown to inhibit Casp1, IL-1β and IL-18 expression through a direct DNA-dependent mechanism in human and mouse primary macrophages (109). In addition, LXRs enhance expression of IL-18BP, the decoy receptor of IL-18, through an indirect IRF8-dependent mechanism (101, 108, 109). In this study, LXRs did not appear to control *Nlrp3* mRNA levels in macrophages. Instead, they inhibit the expression of other inflammasome components such as pro-casp1, pro-IL18 and pro-IL1β and they induce the expression of inhibitory factor including IL18BP. On the contrary, LXR*α* was recently shown to decrease NLRP3 mRNA and protein levels in renal cell carcinomas metastasis *in vivo* and *in vitro*, thus resulting in the reduction of pro-IL1β and pro-caspase1 protein levels and the inhibition of IL1β secretion (144). Finally, lysosomal acid lipase (LIPA)-mediated 25- and 27-hydroxycholesterol (OHC) production, two LXR natural agonists, decreases efferocytosis and metabolic inflammation by activating LXR and by inhibiting NLRP3 in THP1 human macrophages (145). However, the interdependency of each pathway needs further investigations as results from Viaud et al. suggest that 25-OHC dampens inflammasome function independently from LXR activation (145) (**Figure 1**). Instead, it may be due to reduced MAM-dependent mitochondrial repurposing leading to NLRP3 inhibition (145). Therefore, it seems that LXR activity on the NLRP3 inflammasome depends on the cellular and tissular context, underlying cell-specific and context-specific mechanisms that still need to be explained. Interestingly, ERR*α* and PPAR*β*/*δ* increase Mitofusin 2 expression (146, 147). Although the link between the regulation of MAM and NLRP3 has not been established yet, we may anticipate that both NR may be involved in NLRP3 activation.

Epleronone is an antagonist of MR while aldosterone is a MR activator. Interestingly, epleronone-mediated inhibition of MR inhibits IL-1β secretion from eWAT (120). At the molecular level, epleronone treatment prevents ROS production and ATP- or nigericin-induced IL-1β secretion in LPS-primed BMDM, thus suggesting an effect on NLRP3 activation (120). Accordingly, aldosterone induced renal tubular cell injury by activating NLRP3 in a mtROS-dependent manner. Aldosterone-induced IL-1β and IL-18 maturation was then inhibited by NLRP3 knock-down or epleronone-mediated MR inhibition. Epleronone abolishes aldosterone-induced NLRP3, ASC, Casp1, and IL-18 maturation in mouse kidney, but the mechanism is still uncovered (148).

PXR activation with xenobiotics also induces Caspase1 maturation and IL-1β secretion in human THP1 and mouse primary macrophages (149). At the molecular level, PXR promotes rapid ATP release thus acting as an activation signal 2 (149). In this context, SRC kinase (SFK) promotes Pannexin1 phosphorylation thus triggering rapid ATP release (149) (**Figure 1**). It is, however, uncertain whether PXR controls Pannexin1 and SFK at the genomic or non-genomic levels (149). However, since the release of ATP occurs only 15 seconds after PXR agonist stimulation, this effect is unlikely transcriptional but instead it may be due to post-translational modification, advocating for a non-genomic effect of PXR in the regulation of NLRP3 activation step.

Finally, glycolysis and metabolic intermediates were shown to impact NLRP3 activation and ROS production (150). Interestingly, ATRA treatment induces hexokinase 2 expression in human LPS-primed monocyte-derived macrophages, thus shifting the metabolism of macrophages toward glycolysis and activating the NLRP3 inflammasome (122). Imbalance of metabolic homeostasis then appears to be directly linked to the NLRP3 inflammasome activity and the innate immune system thus emphasizing the importance of metabolic sensors in the control of inflammatory pathway. As exemplified here, NRs play an important role in such regulatory processes by bridging metabolism sensing and immunity.

## NLRP3 in the Regulation of NR Activity

Until now, we have reviewed the regulatory effect of NRs on NLRP3. Interestingly, the NLRP3 pathway can also control the activity of NRs. For instance, the NLRP3/Caspase1 complex is able to cleave GR, thus impairing glucocorticoid activity in acute lymphoblastic leukemia (ALL) patients (151). Two cleavage sites of caspase 1, LLID and IKQE, have been identified in GR. Accordingly, increase in caspase 1 induced GR cleavage, decreased GR transcriptional activity and promoted glucocorticoid resistance (151). Interestingly, the comparison of NLRP3 and Caspase 1 expression between glucocorticoid sensitive and resistant primary leukemia cells isolated from 444 patients shows that high expression of Caspase 1 and NLRP3 is associated with an increase in glucocorticoid resistance (151). It is noteworthy that the higher expression of NRLP3 and Caspase 1 observed in glucocorticoid-resistant cells is likely due to lower somatic methylation of their respective promoter (151). Conversely, inhibition of Caspase 1 restores glucocorticoid sensitivity. Similar mechanisms were observed for AR (152).

Finally, the 17-oxo-DHA is a bioactive electrophilic *α,β*-unsaturated keto-derivative of the *ω*3 fatty acid docosahexaenoic acid (DHA) that is endogenously generated by COX2 in activated macrophages (153). The nuclear factor erythroid 2-related factor 2 (Nrf2) is a transcription factor that binds antioxidant response element (ARE) to control antioxidant and detoxifying enzyme transcription including heme oxygenase 1 (HO-1) and glutathione S-transferase (GST). 17-oxo-DHA displays anti-inflammatory and cytoprotective activities by inducing Nrf2-dependent anti-oxidant response and by suppressing NF-*κ*B-dependent inflammatory reactions. Interestingly, 17-oxo-DHA inhibits nigericin-induced ASC speck formation in human THP-1 macrophage cell line. In the context of cigarette smoke-driven chronic obstructive pulmonary disease (COPD), the 17-oxo-DHA compound prevents inflammasome-dependent GR degradation in human peripheral blood mononuclear cells (PBMCs) (153). Although the underlying mechanisms are uncovered, we may speculate that 17-oxo-DHA controls Caspase 1 activity.

## Regulatory Function of NR in NLRP3-Driven Diseases and Their Therapeutic Potential

NLRP3 inflammasome upregulation is involved in numerous inflammatory diseases including joint, intestinal, respiratory, brain, hepatic, kidney, sexual organ and cardiometabolic diseases. Strikingly, NRs were widely involved in the regulation of these diseases through the control of NLRP3 (**Table 1**). It is then not surprising that the modulation of NR activity by specific agonists or antagonists regulates NLRP3 priming or activation and improves or worsens such diseases depending on the context.

**Table 1:** Activity of NRs in NLRP3-driven diseases.

Diseases	NR	Compounds	Effect on inflammasome	Effect on disease	Mechanism****	Reference****
***Brain diseases***						
*Cerebral ischemia*	ER	17β-Estradiol	Inhibition	Neuroprotection	Decreases P2xr7	154, 155
	PPAR*β*/*δ*	GW0742	Inhibition	Decrease neuroinflammation	Decreases TXNIP and NLRP3	134
*Depression*	GR	dexamethasone	Activation	Increase depressive-like behavior	NF-*κ*B-dependent ROS production	116
*Temporal lobe epilepsy*	Rev-erb-*α*, -*β*	SR9009	Inhibition	Preserve neurons	Decreases NLRP3	156
Intestinal diseases						
*Colitis*	FXR	GW4064	Inhibition	Protection	FXR-independent? Inhibition of NF-*κ*B?	125
* *	PPAR*γ*	n/a	Inhibition	Protection	PPAR*γ* mediates naringinin protection	157
* *	VDR	VitD3	Inhibition	Protection	Inhibits NEK7-mediated NLRP3 activation	142
* *	Rev-erb-*α*, -*β*	SR9009	Inhibition	Protection	Decreases NLRP3 in a NF-*κ*B-dependent and independent manner	126
*Colon cancer*	LXR*β*	T091317, GW3945, 25OH-Chst	Activation	Anti-tumoral effect	Interaction with Pannexin-1 and ATP release	143
***Kidney diseases***						
	MR	aldosterone	Activation	Podocyte dysfunction	mitROS production-mediated NLRP3 activation	148, 158, 159
	PPAR*γ*	pioglitazone	Inhibition	Protects renal tubular cells	Inhibits NLRP3 and IL-1β transcription	157
***Respiratory diseases***						
*Acute lung injury*	Rev-erb-*α*, -*β*	SR8278 (antagonist)	Activation	Increases lung water content	Rev-erb inhibition induces NLRP3 inflammasome pathway	127
*P. aeruginosa infection*	PPAR*α*	n/a	Inhibition	Induces complications	Increases NLRP3, ASC, Casp1, and p65 protein level	160
***Cardiometabolic diseases***						
*Atherosclerosis*	LXR	GW3965	Activation	Human study: not defined	IL1-β increases, HIF1*α*-dependent NLRP3 activation (?)	161
*I/R*	Rev-erb	SR9009	Inhibition	Prevents heart failure	Inhibits CCL2 and NLRP3 expression	162
*Diabetic hypertension*	MR	aldosterone	Activation	Increases hypertension and fibrosis	Induces mitROS-mediated NLRP3 activation	163
*Diabetic retinopathy*	PPAR*α*	Fenofibrate	Inhibition	Improves retinopathy	Nrf2-dependent NLRP3 inhibition	164
	Nurr1	n/a	Inhibition	Inhibits Müller glia cells	NF-*κ*B-dependent NLRP3 activation	165
Hepatic diseases						
*Fulminant hepatitis*	Rev-erb-*α*, -*β*	SR9009	Inhibition	Decreases Fulminant hepatitis	Inhibits CCL2/MCP1, NLRP3, IL-18, and IL-1β expression	132
	ROR*γ*	SR1555, SR2211	Inhibition	Decreases Fulminant hepatitis	Inhibits NLRP3 and IL-1β expression	133
*Cholestasis*	FXR	GW4064 (?)	Inhibition	Improves cholestasis-potentiated sepsis	Physically interacts with NLRP3	136
* *	VDR	Calcipotrion	Inhibition	Alleviates cholestatic liver injury	Inhibits NLRP3 pathway and hepatic stellate cell activation	166
*NASH*	PPAR*β*/*δ*	GW501516	Inhibition	Prevents NASH pathogenesis	Inhibits NLRP3, NLRP6, NLRP10, Casp1 and IL-1β expression	167
*I/R*	Rev-erb-*α*, -*β*	SR9009	inhibition	alleviates hI/R-induced hepatic damage	Inhibits NLRP3 and IL-1β expression	168
***Sexual organ diseases***						
*Endometriosis*	ERβ	n/a	Activation	Activate cellular proliferation and adhesion	Inhibits TNF-driven apoptosis and activates NLRP3	169
*Endometrial cancer*	ERβ	Estrogen	Activation	Progression of endometrial cancer	Enhances NLRP3 and IL-1β expression	170

### Brain Diseases

Cerebral ischemia is a particular condition promoting neuroinflammation (154, 155). The 17*β*-Estradiol (E2), an ER agonist, display neuroprotective effect in the context of global cerebral ischemia, a well-known condition in which NLRP3 pathway components are induced (154). In this context, E2 inhibits the expression of NLRP3 inflammasome components and NLRP3 activation by decreasing P2xr7 expression in protein and proline-glutamic acid and leucine-rich protein 1 (PELP1)-dependent manner (154) (**Table 1**). Accordingly, nicotine attenuates ER*β* action on inflammasome activity and exacerbates ischemic brain damage (155). Indeed, nicotine inhibits ER*β* protein levels in hippocampus and cortex while it increases ASC, IL1-β and Caspase 1 protein levels in brain of female rats (155). However, further investigations are needed to demonstrate whether nicotine regulatory effects on the NLRP3 pathway are mediated by ER*β* and NLRP3 instead of a direct activation of the non-canonical or alternative pathway. In addition to ERs, a PPAR*β*/*δ* agonist significantly reduces neuroinflammation after hypoxia–ischemia by inhibiting the expression of TXNIP and NLRP3 (134). Furthermore, temporal lobe epilepsy (TLE) is characterized by spontaneous recurrent seizures leading to neuroinflammation features such as astrocytosis associated with microglia activation and inflammatory cytokine production (156) (**Table 1**). In the context of human and mouse TLE, the Rev-erb ligand, SR9009, prevents neuroinflammation by inhibiting NLRP3 mRNA and protein levels, reducing astrocytes and microglial activation and decreasing apoptosis, which then preserves neurons and provides neuroprotection (156). Finally, glucocorticoids induce NLRP3 in an NF-*κ*B-dependent manner in hippocampal microglial cells, which mediates chronic stress-induced depressive-like behavior in rats (116) (**Table 1**). Altogether, these data demonstrate that NRs such as ERs, Rev-erbs, and GR play a regulatory role on NLRP3-induced brain disease such as cerebral ischemia, epilepsy and depressiveness. As such, the modulation of their activity with ligands may dampen the severity and the progression of such diseases.

### Intestinal Diseases

Colitis is an inflammatory disease of the colon whose causes are still uncertain. We may differentiate acute ulcerative colitis from chronic Crohn’s disease. Strikingly, NLRP3 inflammasome is induced in dextran sulfate sodium (DSS)-induced colitis mouse model. Numerous NRs have then been shown to control DSS-induced colitis severity by modulating NRLP3 inflammasome pathway. For instance, the FXR agonist GW4064 exerts mild effect on colitis reduction by decreasing NLRP3 expression in LPS-induced ileum injury (125) (**Table 1**). However, GW4064 rapidly dampens both canonical and non-canonical NLRP3 activation in an FXR-independent manner, thus questioning the underlying mechanism involved in this fast response (171). Nevertheless, it is not excluded that FXR mediates GW4064 effect after a prolonged exposure to the agonist in this context (172). In obese patients, VDR polymorphisms were associated with increased inflammasome component expression, pro-inflammatory cytokine secretion and gut permeability, or dysbiosis, raising circulating LPS (173). Additionally, VitD3-activated VDR and SR9009-activated Rev-erbs also protect from DSS-induced colitis (126, 142), which then emphasizes the use of such NR-targeted approaches to control inflammatory bowel diseases (**Table 1**). It is noteworthy that numerous compounds derived from Chinese medicine are able to control the inflammasome pathway. For instance, Berberine, isolated from *Rhizoma Coptidis*, has been used for centuries in Chinese medicine to treat gastrointestinal disorders. Intriguingly, Berberine inhibits NLRP3 activation in DSS-induced colitis in a Rev-erb*α*-dependent manner (174). Naringin is a flavonoid extracted from grapefruit, sour orange and citrus seed that display anti-inflammatory properties (175). Interestingly, PPAR*γ* mediates the anti-inflammatory effects of Naringin on DSS-induced ulcerative colitis (175). Whether thiazolidinediones, a PPAR*γ* agonist class, prevent colitis progression as well remains to be determined (**Table 1**). Finally, LXRβ activates NLRP3 inflammasome in colon cancer cells leading to anti-tumoral effect of LXR agonists (143) (**Table 1**).

### Kidney Diseases

Podocytes are important glomerular cell types playing a key role in blood filtration by the kidney. Aldosterone, a MR agonist, drives NLRP3-dependent podocyte dysfunction *in vivo* and *in vitro* by inducing oxidative stress (158) (**Table 1**). Remarkably, eplerenone, that inhibits MR, protects podocytes from aldosterone-induced injury (158). However, the dependency of MR in this context still needs to be addressed. In addition to podocytes, aldosterone also induces renal tubular cell injury. These cells play a pivotal role in the absorption of glucose, amino acids and ions by the renal tubule. In this context, aldosterone promotes mtROS production and subsequent NLRP3 activation (159). Strikingly, aldosterone induces NLRP3, IL1β, IL18 and CASP1 expression in human immortalized normal kidney cells isolated from proximal tubules (HK-2 cells) in a dose- and time-dependent manner, thus inducing a phenotypic switch from HK-2 to fibroblast/pericyte cells in a MR and NLRP3-dependent manner (159). Accordingly, eplerenone abolishes these aldosterone-mediated effects. In addition, NLRP3 deletion in mice attenuates aldosterone-induced renal injury by protecting cells from apoptosis/pyroptosis and by preventing this phenotypic switch (159). Finally, aldosterone also induces tubulointerstitial fibrosis leading to kidney failure (148). As above, eplerenone abolishes aldosterone-induced macrophage infiltration, tubulointerstitial fibrosis in a MCP1- and ICAM1-dependent manner (148). Precisely, macrophage inflammasome was required to induce renal fibrosis and kidney dysfunction after aldosterone administration, whereas renal cells were involved in MCP1 expression, showing a cell-specific aldosterone action in renal failure. However, the dependency on MR in renal fibrosis is still elusive (148). Finally, PPAR*γ* activation with pioglitazone inhibits MSU-induced NLRP3 and IL-1β mRNA and protein levels in HK-2 cells (157) (**Table 1**). Intriguingly, MSU and LPS were able to induce PPAR*γ* expression in HK-2 cells after a short exposure, but not a long exposure, thus suggesting that PPAR*γ* sets up a negative feedback loop to inhibit NLRP3 activation (157).

### Respiratory Diseases

Acute lung injury is a severe IL-1β-associated complication that occurs after pulmonary inflammation and increases the mortality rate in patients. In mice, the Rev-erb antagonist SR8278 exacerbates LPS-induced lung permeability, which increases lung water contents (127) (**Table 1**). In this context, SR8278 increases macrophage recruitment in the lung and enhances IL-1β production in bronchioalveolar lavage fluid (127). In addition, PPAR*α* ablation in mice increases NLRP3, ASC, Caspase 1 and p65 protein levels in the lung after infection with *Pseudomonas aeruginosa* (PA), which then promotes lung complications and subsequently worsens the pathophysiology of PA lung diseases (160) (**Table 1**).

### Cardiometabolic Diseases

Cardiometabolic diseases include hypertension, diabetes, non-alcoholic fatty liver diseases, vascular dysfunction, and heart failure. They share common inflammatory features including NLRP3 inflammasome activation. We may distinguish atherosclerosis, heart ischemia–reperfusion, obesity, type 2 diabetes, and diabetic retinopathy. Atherosclerosis is a lipid-driven inflammatory disease of the vascular wall during which infiltrating LDLs are eventually modified, triggering their uptake by macrophages. Oxidized LDLs are indeed internalized and promote both priming and cholesterol crystals-mediated activation of NLRP3 in a CD36-dependent manner (19, 20). Accordingly, ablation of the NLRP3 inflammasome pathway decreases atherosclerosis progression (176, 177). Numerous NRs have been shown to be involved in atherosclerosis development including PPARs, Rev-erb*α*, LXRs, and Nur77 (3, 86, 108, 178). Interestingly, an LXR agonist has lately been shown to increase IL-1β protein levels in an HIF1*α*-dependent manner in human atherosclerotic lesions. It is however unknown whether it relies on an LXR-dependent mechanism (161) (**Table 1**). However, as HIF1*α* induces NLRP3 inflammasome activation (179–181), such regulatory mechanism may then account for LXR-dependent activation of IL1-β production in hypoxic atherosclerotic lesions.

Diabetes and hypertension are common coexisting diseases that accelerate micro and macrovascular complication occurrence. Different groups evidenced that aldosterone-activated MR increases hypertension and fibrosis through mtROS-mediated NLRP3 activation (163). In a model of obese diabetic *db/db* mice, spironolactone-mediated MR inhibition ablates inflammasome activation in mesenteric arteries (163) (**Table 1**). In addition, spironolactone treatment ameliorates glucose homeostasis without affecting body mass and mesenteric artery KCl-induced contraction. However, spironolactone ameliorates acetylcholine-activated vasorelaxation in phenylephrine-contracted mesenteric artery *ex vivo* (163). Accordingly, the NLRP3 inhibitor MCC950 mimics spironolactone effect in this vasoreactivity model, then suggesting that NLRP3 controls vasoreactivity in a MR-dependent manner (163). Diabetic retinopathy is a common neurovascular complication of diabetes that represents the most frequent cause of vision loss and blindness worldwide. In early non-proliferative stages, hyperglycemia causes glucotoxicity and damages retinal small vessels. As the disease progresses, alteration of small vessels triggers hypoxia and the development of small, fragile neovessels that can bleed, clot, and alter the retina. Because of cell death, diabetic retinopathy may also be considered as a chronic low-grade inflammatory disease in which the NLRP3 inflammasome is activated (54, 182). Strikingly, treatment with the PPAR*α* ligand fenofibrate (FF) ameliorates diabetic retinopathy by inducing Nrf2 signaling and inhibiting NLR3 inflammasome (164) (**Table 1**). FF inhibits Nrf2 expression in mouse retinal Müller glial cells and attenuates gliosis in diabetic retina (164). However, it is uncertain whether FF effect is mediated by PPARα activation. Finally, Nurr1 deficiency promotes high glucose-induced Müller glial cell activation by inducing NF-*κ*B and the NLRP3 inflammasome axis (165) (**Table 1**).

Post-ischemia reperfusion (I/R), after a heart ischemic episode, triggers a profound inflammatory response called reperfusion injury, which provokes adverse cardiac remodeling and heart failure. Consistently, MCC950-mediated NLRP3 inhibition lowers infarct size and areas at risk (183). Remarkably, administration of SR9009 Rev-erb agonist, one day after myocardial I/R, prevents heart failure by targeting cardiomyocyte inflammasome in a Rev-erb-dependent manner (162) (**Table 1**). In addition, Rev-erb activation inhibits CCL2 secretion and leucocyte recruitment at ischemic sites, thus lowering cardiac inflammation that would prevent cardiac remodeling (162).

### Hepatic Diseases

Cholestasis is a common liver complication in patients with extrahepatic infection or sepsis and consists in bile acid accumulation in liver and serum. Intriguingly, on the one hand, BAs behave as DAMPs which activate both priming and activation of NLRP3, while on the other hand, the BA receptor FXR inhibits NLRP3 activation by physically interacting with NLRP3 (136) (**Table 1**). However, because the GW4064 compound may modulate NLRP3 activity in an FXR-independent manner (171), we may anticipate that BA effects on NLRP3 in cholestatic mice may also occur in an FXR-independent manner, thus explaining this apparent discrepancy. However, as FXR expression is down-regulated in endotoxic mice, FXR synthetic ligands display a poor effect on cholestasis (136), thus advocating for the identification of an alternative therapeutic strategy such as promoting the increase of FXR expression. Finally, the VDR agonist calcipotriol is also able to alleviate cholestatic liver injury and fibrosis by inhibiting the NLRP3 inflammasome pathway involved in inflammation, and hepatic stellate cells activation likely responsible of fibrosis (166) (**Table 1**).

Non-alcoholic fatty liver diseases (NAFLD) are common chronic liver diseases, ranging from hepatic steatosis to non-alcoholic steatohepatitis (NASH), which is characterized by lipid accumulation, inflammation, and fibrosis (184). NASH may eventually progress to irreversible cirrhosis and hepatocarcinoma (184). Remarkably, inhibition of the NLRP3 inflammasome pathway reduces liver inflammation and fibrosis in an experimental mouse NASH model (185). Interestingly, the dual PPAR*α* and PPAR*β*/*δ* agonist GFT505/Elafibranor displays hepatoprotective effects in different rodent models of NASH by reducing fibrosis and cytokine secretion including IL-1β (167). Consistently, administration of the PPAR*β*/*δ* agonist GW501516 inhibits Caspase 1 and IL-1β hepatic mRNA levels in mice fed a high fat diet (HFD) and co-treated with LPS (186) (**Table 1**). In human hepatic hepG2 cell line, palmitic acid and LPS co-treatment induces the expression of NLRP3, NLRP6 and NLRP10 as well as Caspase 1 and IL-1β (186). Consistently with *in vivo* data, GW501516 prevents palmitate/LPS-induced inflammasome component gene expression (186). Intriguingly, although GW501516 accordingly impairs Caspase 1 maturation, it does not control IL-1β secretion (186).

Fulminant hepatitis (FH) is a life-threatening condition characterized by fast evolving hepatic dysfunction associated with tissue necrosis, inflammation and hepatic encephalopathy (187). Albeit numerous factors including fungi intoxication, viral infection, and metabolic diseases trigger FH, the main cause of FH nowadays is drug overdose with acetaminophen as the main one (187). Acetaminophen accumulation induces P450-mediated overproduction of toxic metabolites leading to oxidative stress, mitochondrial membrane potential loss and hepatocellular death. Tissue necrosis is then responsible of the release of DAMPs such as ATP and subsequent NLRP3 inflammasome activation (188, 189). Strikingly, *Rev-erbα*-deficiency aggravates FH in a mouse model of LPS-galactosamine (GalN)-induced liver injury. This occurred in an NLRP3-dependent manner by alleviating its inhibitory effect on Caspase 1 activity and on IL-1β expression and secretion (132) (**Table 1**). As Rev-erb*α* also impairs CCL2/MCP1 chemokine expression, ablation of Rev-erb*α* worsened neutrophils and monocytes infiltration in LPS/GalN-challenged mice, thus contributing to increased liver injury (132). Consistently, pre-treatment with the Rev-erb agonist SR9009 prevents LPS/GalN-induced FH pathogenesis by inhibiting the NLRP3 inflammasome pathway and CCL2 expression, thereby delaying death and improving the survival rate from 10% in the control to 70% in the SR9009-treated mice (132). Finally, the ROR*γ* inverse agonists SR1555 and SR2211 reduce the expression and secretion of IL-1β in LPS/GalN-induced FH and exert a hepatoprotective effect that improves the survival rate of treated FH mice (133) (**Table 1**). However, whether ROR*γ* mediates SR1555 and SR2211 effect on NLRP3 pathway and FH protection still needs to be proven. Nevertheless, ROR*γ* deletion in LPS-primed BMDM inhibits NLRP3 and IL-1β secretion, which is consistent with a ROR*γ*-inhibiting effect of SR1555 and SR2211 on these processes (133).

Rev-erb-*α* has also been highlighted lately in the context of hepatic ischemia–reperfusion (hI/R). hI/R is a complex phenomenon during which hepatocyte damage hits when blood supply returns into the ischemic liver after a liver transplantation, hepatectomy, and ischemic shock (190). Inflammatory responses play an important role in hI/R injury during which activated Kupffer cells release ROS and pro-inflammatory cytokines including IL-1β. Consistently, NLRP3 deficiency protects against liver I/R injury in mice (191). Accordingly, *Rev-erbα* deletion sensitizes mice to hI/R and is accompanied by exacerbated NLRP3 activation and pro-inflammatory cytokine secretion (168). On the contrary, SR9009 treatment alleviates hI/R-induced hepatic damage by inhibiting IL-1β expression (168). In conclusion, Rev-erbs, ROR*γ*, VDR, PPAR*β*/*δ*, and FXR then exhibit hepatoprotective effects in acute liver inflammatory diseases by dampening the NLRP3 inflammasome activity.

### Sexual Organ Diseases

Endometriosis is a sexual organ disease originating from abnormal deposition of endometrial cells that grow outside from the uterine cavity. It affects 6–10% of reproductive-aged women. Endometriosis causes pelvic pain in 50% of cases and fertility problem in 40–50% of cases (169). Endometriosis is likely due to high production levels of 17*β*-estradiol that could play a role in the proliferation of endometriotic tissues (169) (**Table 1**). Compared to ER*α*, ER*β* expression is significantly higher in endometriotic tissue than in normal uterine endometrium in human. In addition, the role and the specific expression of ER*α* in endometriotic tissues are controversial (169). Interestingly similar patterns were observed in the mouse (169). Interestingly, *NLRP3^−/−^* mice exhibit smaller ectopic lesions compared to wild type mice, thus suggesting that NLRP3 induces endometriosis (169). Strikingly, ERβ inhibits TNFα-driven apoptosis and activates NLRP3 in endometriotic tissues (169). Accordingly, ER*β* then increases IL-1β secretion, which enhances cellular adhesion and proliferation (169). Consistently, NLRP3 inflammasome activation has been shown to promote the progression of human endometrial cancer in an ER*β*-dependent manner (170) (**Table 1**). At the molecular level, ER*β* interacts with the NLRP3 inflammasome in the cytoplasm (169). However, the exact regulatory mechanism still needs to be investigated.

## NR-Dependent Control of NLRP3 Circadian Rhythmicity and Chronotherapy

Our ability to anticipate environmental changes imposed by the rotation of the Earth is controlled by the circadian clock, which properly gates many, if not all, physiological processes to the most appropriate time window (192). Among these physiological pathways, immune functions vary according to the time of day (3, 193), a process described as circadian immunity, in which innate immune cells such as macrophages harbor an intrinsic clockwork that drives circadian transcription of genes involved in the response to bacterial challenge (105, 194, 195). Pioneer studies have demonstrated that important features of the immune system such as trafficking and abundance of blood leucocytes, their recruitment to tissue, their ability to respond to pathogens and to secrete immune molecules vary in a circadian manner (196, 197). At the molecular level, the biological clock is a complex network of transcription factors and interlocked transcriptional feedback loops that orchestrate cellular circadian rhythms. Among the core clock components, the ligand-activated nuclear receptors Rev-erbs and RORs participate in the circadian control of the immune system (104, 106), whereas pharmacological activation of Rev-erb*α* and ROR modulates the expression and release of key pro-inflammatory cytokines (105, 133). It is noteworthy that Rev-erb nuclear receptors, altogether with ROR*α* are the only core clock components whose activity may be directly modulated by a synthetic compound, thus representing an interesting therapeutical approach to directly modulate immune circadian behavior (198).

Over the past 100 years of global industrialization, mankind underwent some important changes in its lifestyle including its food habits, the ease of travel, the increase in shift work and social demands, and erratic exposure to artificial light from luminescent screens, which have dramatically altered circadian rhythms. It is now well-recognized that disruption of the intrinsic molecular clock impedes a proper immune response (199) and has severe repercussions on health. Indeed, numerous clinical studies have demonstrated that disruption of circadian rhythms in human represents an additional risk factor for neurological, metabolic and chronic inflammatory disorders (200–202) such as asthma, rheumatoid arthritis, atherosclerosis, type 2 diabetes or Alzheimer Disease (193, 202). Most of the clock-driven diseases demonstrate a chronic inflammatory component, either infiltration of macrophages in the vascular wall due to an accumulation of non-infectious DAMPs such as cholesterol crystal causing atherosclerosis, hydroxyapatite in joints leading to rheumatoid arthritis or the deposit of β-amyloid fibers, which activates microglial cells in Alzheimer disease (197).

Remarkably, clock disruption alters NLRP3 circadian oscillations in a mouse model of jetlag or in genetic and pharmacological models of clock alteration, thus modulating the progression of inflammatory diseases (3) including colitis (126), myocardial infarction/ischemia–reperfusion injury (162), lung injury (127) and fulminant hepatitis (132, 133). At the molecular level, NLRP3 expression altogether with IL-1β and IL-18 mRNA levels oscillate in a daily manner under the control of Rev-erb*α in vivo* and *in vitro* (132). Indeed, Rev-erb*α* ablation abolishes circadian oscillations in *Nlrp3* gene expression in peritoneal macrophages and in serum shock-synchronized human and mouse primary macrophages, with functional repercussions on IL-1β and IL-18 oscillatory secretion (132). *In vitro*, Rev-erb*α* deletion promotes elevated expression of *Nlrp3*, *Il1β* and *Il18*, which is accompanied by an increase in IL-1β and IL-18 secretion (132). By contrast, activation of Rev-erbs with heme, their natural ligand, or with synthetic ligands reduces the secretion of these cytokines by inhibiting the expression of NLRP3 inflammasome component genes (132). Strikingly, the susceptibility to fulminant hepatitis and hepatic ischemia reperfusion injury is time-of-day dependent, upon the control of the molecular clock with Rev-erbα as an important regulator of the inflammasome (132, 168). Remarkably, pharmacological activation of both Rev-erbs and ROR reduces liver injury and improves the survival time and rate in a NLRP3-dependent manner in treated mice (132, 133). Consistently, time of cardiac ischemia/reperfusion and subsequent SR9009 treatment affect heart function recovery, the best response being obtained when Rev-erb expression is at its highest, *ie* when NLRP3 expression is at its lowest (162). Interestingly, the NF-*κ*B-driven long non-coding RNA *Lnc-UC* has lately been shown to be induced by the core clock component Bmal1, thereby generating circadian expression of *Lnc-UC* (203). Then, *Lnc-UC* physically interacts with Cbx1 protein to reduce its gene silencing activity *via* H3K9me3, thereby enhancing Rev-erb*α* expression in an epigenetic manner (203). Then, by inducing Rev-erb*α* expression, *Lnc-UC* ablates NF-*κ*B signaling and NLRP3 inflammasome signaling in macrophages (203). Consistently, *Lnc-UC* deletion disrupts clock gene expression, sensitizes mice to DSS-induced colitis and disrupts the diurnal rhythmicity in disease severity (203). Additionally, Rev-erb*α*-mediated effect of Berberine on DSS-induced colitis shows better effect when administered at ZT10 (late resting phase) compared to ZT2 (early resting phase), thus acknowledging the rationale to target core clock components in the control of NLRP3-driven diseases (174). Such circadian effect of drug efficiency might be explained by the lower severity of colitis at ZT10, which coincides with the maximum expression of Rev-erb*α*. In conclusion, circadian pharmacological effects of compounds on different diseases likely result from diurnal rhythms of both disease severity and daily oscillations of the drug target expression. Altogether, these observations advocate for chronotherapeutic practice on NLRP3-driven diseases.

## Concluding Remarks

NLRP3 inflammasome deregulation drives numerous diseases. Inhibition of NLRP3 using MCC950 demonstrates beneficial effects in fulminant hepatitis and in myocardial ischemia reperfusion (3). However, MCC950 displays hepatotoxic properties advocating for the development of alternative NLRP3 inhibitory strategies (3). Here, we provide the first extensive review showing the close links between nuclear receptors and the NLRP3 inflammasome pathway. Indeed, NRs are able to either activate the NLRP3 inflammasome or inhibit both priming and activation steps of the NLRP3 inflammasome pathways, acting at different levels, which offers numerous possibilities to modulate NLRP3-driven disorders. Indeed, the activity of NRs can be modulated by a plethora of synthetic, but also natural ligands. As such, NRs should be considered as sensors of environment changes including metabolic alterations, hormonal signal, pollutions and circadian rhythmicity. As NRs are able to control similar processes, we may consider that the entire NR family integrate these different environmental modifications, that may occur simultaneously, to deliver the best response. We may then anticipate that depending on their environment, NRs cooperate to appropriately modulate the NLRP3 inflammasome. NRs would then allow the adaptation of the innate immune system and the NLRP3 inflammasome to adjust its response from cytokine secretion to pyroptosis-induced cell death. Finally, nuclear receptors, including Rev-erb and ROR, control the circadian expression of NLRP3. As such, NLRP3 protein amounts are not equal across the day, thereby emphasizing the necessity of a chronotherapeutic approach. In the case of clock disruption as observed in shift workers or in elderlies for instance, targeting clock components to re-entrain the molecular clock and sustain circadian amplitude of NLRP3 expression may also be considered as an alternative or an additional approach.

## Author Contributions

BP and HD wrote and edited the manuscript and the figures. All authors contributed to the article and approved the submitted version.

## Funding

Some of our work included in this review manuscript was supported by the Fondation pour la Recherche Médicale (FRM, EQU202003010310), INSERM, the ANR-Labex-EGID (EGID, ANR-10-LABX-46), the Fondation Francophone pour la recherche sur le diabète (FFRD) together with the Fédération Française des Diabétiques (AFD) AstraZeneca, Eli Lilly, Merck Sharp &amp; Dohme (MSD), Novo Nordisk &amp; Sanofi; the Région Hauts-de-France/FEDER (Chronoregeneration), Association Française contre les Myopathies (AFM), Fondation de France, Société Francophone du Diabète (SFD)-SERVIER and the ANR. This project is cofounded by the European Union under the European Region Development Fund (ERDF) and by the Hauts de France Region Council (contract_20000007), the MEL (contract-2020-ESR-02), and the French State (contract n°2019-R3-CTRL_IPL_Phase3). This project is cofounded by the European Union under the European Region Development Fund (ERDF) and by the Hauts de France Region Council (contract_20002842), the MEL (contract-2020-ESR-06), and the French State (contract n°2020-R3-CTRL_IPL_Phase4). This project is co-funded by the Agence Nationale pour la Recherche (ANR) (ANR-19-CE15-0033-01). The funders were not involved in the study design, collection, analysis, interpretation of data, the writing of this article or the decision to submit it for publication.

## Conflict of Interest

The authors declare that the research was conducted in the absence of any commercial or financial relationships that could be construed as a potential conflict of interest.
